# Anomeric Spironucleosides of β-d-Glucopyranosyl Uracil as Potential Inhibitors of Glycogen Phosphorylase

**DOI:** 10.3390/molecules24122327

**Published:** 2019-06-25

**Authors:** Aggeliki Stathi, Michael Mamais, Evangelia D. Chrysina, Thanasis Gimisis

**Affiliations:** 1 Organic Chemistry Laboratory, Department of Chemistry, National and Kapodistrian University of Athens, 10571 Athens, Greece; iamaggeliki@yahoo.gr (A.S.); mmamais@chem.uoa.gr (M.M.); 2 Institute of Chemical Biology, National Hellenic Research Foundation, 11635 Athens, Greece; echrysina@eie.gr

**Keywords:** type 2 diabetes, glycogen phosphorylase, anomeric spironucleosides, 1,6-dioxa-4-azaspiro[4.5]decane, [1,5]-radical translocation

## Abstract

In the case of type 2 diabetes, inhibitors of glycogen phosphorylase (GP) may prevent unwanted glycogenolysis under high glucose conditions and thus aim at the reduction of excessive glucose production by the liver. Anomeric spironucleosides, such as hydantocidin, present a rich synthetic chemistry and important biological function (e.g., inhibition of GP). For this study, the Suárez radical methodology was successfully applied to synthesize the first example of a 1,6-dioxa-4-azaspiro[4.5]decane system, not previously constructed via a radical pathway, starting from 6-hydroxymethyl-β-d-glucopyranosyluracil. It was shown that, in the rigid pyranosyl conformation, the required [1,5]-radical translocation was a minor process. The stereochemistry of the spirocycles obtained was unequivocally determined based on the chemical shifts of key sugar protons in the ^1^H-NMR spectra. The two spirocycles were found to be modest inhibitors of RMGP*b*.

## 1. Introduction

**Figure 1:**
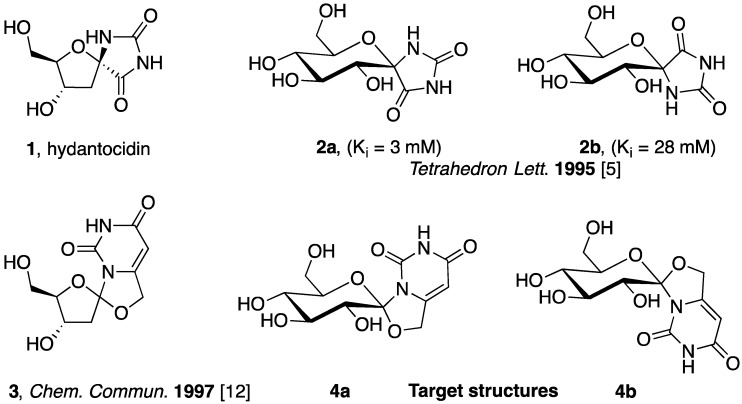
Natural and synthetic anomeric spironucleosides.

Despite the prevalence of type 2 diabetes worldwide, no sufficient treatment has been identified; therefore, a molecular approach based on the three-dimensional structure of enzymes directly involved in glycogen metabolism has received increasing attention. Glycogen phosphorylase (GP) is an allosteric enzyme with a regulatory role in glycogen breakdown to glucose [1]. Since glucose is the physiological substrate of GP, it promotes the inactive form of the enzyme acting synergistically with insulin towards reducing the rate of glycogen degradation and shifting the equilibrium towards glycogen synthesis. GP three-dimensional structure in the T state (GPb) has been exploited as a target for the design of glucose-based compounds that inhibit enzymic action preventing glycogenolyis and acting as regulators of glucose levels in the bloodstream [2,3]. The glucose specificity for the catalytic site of the enzyme has been utilized to drive glucose derivatives to the active site of the enzyme, exploiting the catalytic channel by adding a variety of structural features to these compounds in terms of rigidity and functional groups [4]. One of the early lead inhibitors of rabbit muscle glycogen phosphorylase *b* (RMGP*b*) was pyranosyl spironucleosides [5] (**2a**,**b**, Figure 1), the structure of which was inspired from hydantocidin (**1**), a natural spiro compound with herbicidal and plant growth regulatory activity. To this end, a number of attempts to synthesize more potent inhibitors were made leading to other spiro-heterocycles that exhibited stronger affinity for RMGP*b* [6]. Similar studies in our laboratory led to *N*^4^-aryl-*N*^1^-(β-d-glucopyranosyl)cytidine nucleosides which exhibit RMGP*b* inhibition in the nanomolar range [7].

We present here a methodology, that involves a key [1,5]-radical translocation step for the synthesis of anomeric spironucleosides **4a**,**b** (Figure 1), which were found to be modest inhibitors of rabbit muscle glycogen phosphorylase (RMGP*b*). Spirocyclic nucleosides present a rich synthetic chemistry and important biological function [8,9]. The targets in this paper contain a rare 1,6-dioxa-4-azaspiro[4.5]decane structure [10], which has not been previously constructed via a radical pathway [11]. In the similar 1,6-dioxa-4-azaspiro[4.4]nonane system (**3**, Figure 1), we reported an efficient protocol, using a 6-lithiation strategy for generating a 6-hydroxymethyluridine intermediate followed by oxidative cyclization through a [1,5]-radical translocation strategy [12,13]. We were interested in applying this protocol to the related “decane” system.

## 2. Results

### 2.1. Synthesis

**Scheme 1:**
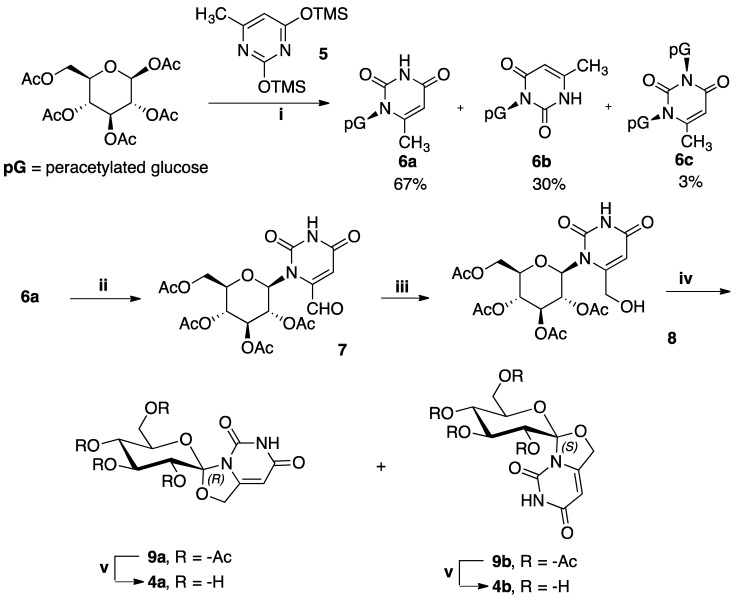
(**i**) TMSOTf, DCE, reflux, 1 h, 67%, (**ii**) SeO_2_, dioxane, AcOH, reflux, 5 h, 67%, (**iii**) NaBH_4_, silica gel, CHCl_3_, propanol, −30 °C, 1 h, 90%, (**iv**) DIB, I_2_, CH_2_Cl_2_, hv, r.t., 2.5 h, 18% (9a:9b = 1.25:1), and 50% (**7**), (**v**) NH_3_ (7N in MeOH), r.t., 16 h, 100%.

Initially, we attempted to access compound **7** (Scheme 1) by direct lithiation of the known 2,3,4,6-tetra-*O*-benzyl-β-d-glucopyranosyluracil [14]. The protocol was based on the previous well-established 6-lithiation of protected 2-deoxy- and ribouridines, followed by the reaction with dimethylformamide or ethyl formate to generate the corresponding 6-formyluridines [12,15,16]. Although a major product formed under these conditions, spectral analysis revealed that it was the product of a selective mono-debenzylation and did not contain a formyl group. Although we could not unequivocally determine the position of debenzylation, we hypothesized that this had occurred in position-3, proximal to the 6-position where the initial lithiation is expected to occur (data not shown). This reductive debenzylation could be reminiscent of a previous method employing lithium naphthalenide [17].

The above result prompted us to change our strategy and include a previously reported [18] *N*^1^-(β-d-glucopyranosyl)-6-methyluracil (**6a**) intermediate in our synthesis, as exemplified in Scheme 1. Under optimized conditions, the *N*-glycosylation reaction of persilylated 6-methyluracil, in the presence of an excess of TMSOTf in DCE, led to the formation of three products, namely, **6a**–**c**, isolated after column chromatography in 67%, 30%, and 3% yield, respectively. As determined by ESI-MS and NMR, the major product was the expected *N*^1^-glycosylated 6-methyluridine **6a**, whereas the *N*^3^-glycosylated analogue **6b** was isolated in 30% yield, together with a small amount of *N*^1^,*N*^3^-bisglycosylated isomer **6c**.

The main feature that differentiated **6b** from **6a** in the ^1^H-NMR spectrum was a substantial downfield shift of H-2′ (δ∆ = 0.8 ppm) in **6b**, induced by the magnetic anisotropy effect of the second vicinal amidic 4-carbonyl. A similar effect was observed in the more complex spectra of the bis-substituted analogue **6c**, and the [Μ + H]^+^ peak at 786.3 amu (ESI-MS) clearly differentiated **6c** from the other two isomers ([M + H]^+^ at 457.2 amu).

The allylic methyl group of **6a** was oxidized to the corresponding aldehyde **7** in the presence of selenium dioxide (3 equivs) in dioxane: acetic acid [19], in 67% yield. Apart from the aldehyde **7**, isolated in 67% yield and recognized in ^1^H-NMR by its characteristic aldehydic proton at 9.90 ppm, a second more polar product was isolated in low yield, identified as the allylic alcohol **8**. Its formation is expected by the mechanism of the reaction which follows an electrophilic allylic addition to selenium followed by a [2,3]-sigmatropic rearrangement [20,21]. Application of stoichiometric amounts of SeO_2_ also led to aldehyde **7** as the major product, although full conversion was not observed even after prolonged reaction times. When the reaction was performed with 3 equivs of SeO_2_, under strictly anhydrous conditions, the yield of aldehyde **7** was maximized. Reduction of aldehyde **7** with NaBH_4_ in CHCl_3_/isopropanol, in the presence of silica gel, at 0 °C [22] led to partial removal of acetate groups. By lowering the reaction temperature to −30 °C, exclusive formation of the allylic alcohol **8** was observed, and the product was isolated in 90% yield.

The key step photolysis of **8**, under the standard optimized Suárez conditions [23], utilized for alkoxy radical generation in hydrogen atom transfer (HAT) reactions [11], in the presence of DIB and I_2_, in DCM and under visible light (150 W) irradiation, led to the isolation of three products in 50%, 10%, and 8% yield. The major product was, surprisingly, the aldehyde **7**, whereas the two minor products corresponded to the expected isomeric spironucleosides **9a**,**b**. The reaction is expected to proceed through the generation of an alkoxyl radical intermediate, followed by a [1,5]-radical translocation [24] to generate a C-1′ radical intermediate which, after oxidation and ionic cyclization, provides the spirocycles **9a**,**b** [12,13]. The main formation of aldehyde **7** can be explained by a possible disproportionation reaction of the above alkoxy radical intermediate to aldehyde **7** and alcohol **8**, with the latter re-entering the reaction cycle (see discussion below). The application of the same conditions as in the similar 1,6-dioxa-4-azaspiro[4.4]nonane system [12,13] aids in the comparison of the two systems and indicates that the rigidity of the β-d-glucopyranosyl ring renders the [1,5]-hydrogen atom transfer less favorable in this system than in the previously observed flexible ribosyl system. It should be noted that when the same reaction was attempted with a similar photolysis in the presence of Pb(OAc)_4_, I_2_, and CaCO_3_ [25], a complex mixture of products was obtained that could not be further characterized. Final removal of the acetate protection of **9a,b** with ammonia in methanol led to the isolation of the target spirocycles **4a**,**b** in quantitative yield.

### 2.2. Kinetic Experiments

RMGP*b* was isolated, purified, and recrystallized according to previously established protocols [26]. Compounds **4a**,**b** were assayed in the direction of glycogen synthesis for their inhibitory effect on RMGP*b* as described before [26,27]. They both exhibited competitive inhibition with respect to the substrate glucose 1-phosphate (Glc-1-P), at constant concentrations of glycogen (0.2% *w*/*v*) and AMP (1 mM). Compound **4b** was found to be a stronger inhibitor of RMGP*b* (35% inhibition at 1 mM) than **4a** (26% inhibition at 1 mM).

## 3. Discussion

**Figure 2:**
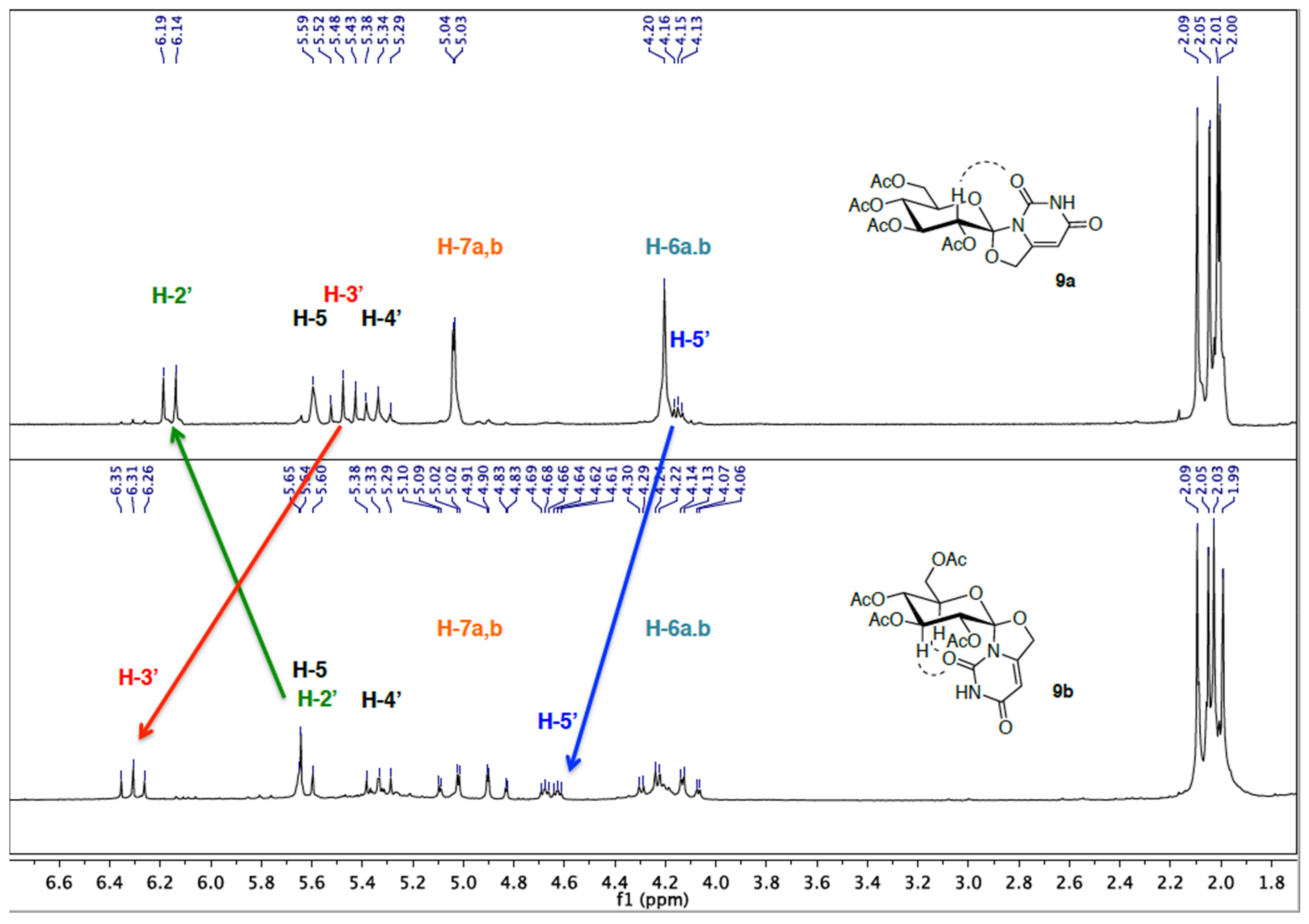
Comparison of ^1^H-NMR spectral shifts in compounds **9a**,**b**.

The stereochemistry of the two spirocyclic products could be inferred unequivocally, from the ^1^H-NMR spectra, as can be seen in Figure 2. Both spectra contain features that can be explained by the magnetic anisotropy induced by the 2-C=O group onto the sugar α- or β- hydrogens depending on the stereochemistry of the new spiro-center. As the new spirocycle locks the configuration of the pyrimidine ring with respect to the sugar ring, the 2-C=O is spaced in the vicinity of H-2′ in the *R*-anomer **9a** and on the other hand, in the vicinity of H-3′ and H-5′, in the *S*-anomer **9b**. This results in significant downfield shifts of the corresponding sugar Hs in the ^1^H-NMR spectra. Specifically, there is a 0.55 ppm shift of H-2′ going from the *S*- to *R*-anomer (5.62 to 6.17 ppm), whereas there is a 0.48 and 0.84 ppm shift for H-5′ (4.18 to 4.65 ppm) and H-3′ (5.47 to 6.31 ppm), respectively, going from the *R*- to the *S*-anomer (Figure 2). The remaining Hs (H-4′, H-6′, H-5, and H-7) have similar chemical shifts in the two spectra, with the difference that the pairs of H-6′ and H-7 protons appear as AB quartets in the case of the more congested *S*-anomer **9b**, whereas they collapse to singlets in the case of the *R*-anomer **9a**.

The same trends reported above for the protected derivatives were also observed in the case of the final compounds **4a**,**b**. Specifically, there was a 0.61 ppm shift of H-2′ going from the *S*- (**4b**) to *R*- (**4a**) anomer (3.95 to 4.56 ppm), whereas there is a 0.37 and 0.79 ppm shift for H-5′ (3.90 to 4.27 ppm) and H-3′ (3.83 to 4.62 ppm), respectively, going from the *R*- (**4a**) to the *S*- (**4b**) anomer. The remaining Hs (H-4′, H-6′, H-5, and H-7) had similar chemical shifts in the two spectra, with the difference, again in this case, being that the pairs of H-6′ and H-7 protons appear as AB quartets in the case of the more congested *S-*anomer **4b**, whereas they collapse to singlets in the case of the *R-*anomer **4a**. It should be noted that 2D NOESY spectroscopy did not provide any additional information for the above systems, as the only correlations that were observed were those between either protons H-2′ and H-4′ or H-3′ and H-5′, above and below the plane of the glucopyranosyl ring, respectively.

The above analysis allowed us to better interpret the ^1^H-NMR spectrum of compound **8** (in DMSO-d6) which appeared as a mixture of rotamers, indicating a slow, on the NMR time scale, rotation around the glycosidic C^1^′-N^1^ bond, due to the new 6-hydroxymethyl substituent. The existence of rotamers was indicated by the presence of two amidic hydrogens at 11.48 and 11.32 ppm in a ≈1:2 ratio. Two more characteristic low field signals were a doublet at 6.43 ppm and a triplet at 6.08 ppm exhibiting the same ≈1:2 ratio. By applying the analysis above, performed for the final spirocyclic products, one can assign the doublet to the H-2′ of the rotamer with the 6-CH_2_OH group in α-position and the triplet to the H-3′ of the conformer with the 6-CH_2_OH group in β-position. When the ^1^H-NMR spectrum of **8** was obtained at a higher temperature (75 °C), the above signals collapsed to broad singlets, confirming the above hypothesis. The observed ratio of the two rotamers at equilibrium is significant in the next key step, as explained below.

**Scheme 2:**
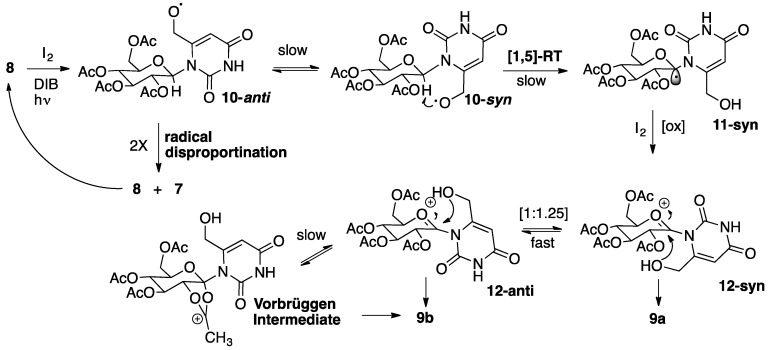
Proposed mechanism for the formation of **9a**,**b**.

Regarding the mechanism of the key step, it is expected that, under the Suárez conditions, an alkoxy radical intermediate is produced that may exist in two possible conformations (**10-syn**, **10-anti**, Scheme 2). These two conformers are similar to those observed above for alcohol **8** and are expected to be formed in a similar ≈1:2 ratio (**10-syn**:**10-anti**). The rigidity of the pyranosyl chair conformation may not allow a fast interconversion between the conformers for steric reasons, and the product distribution may be affected directly by these two conformer populations and their corresponding reactivity. Specifically, **10-anti** cannot undergo a [1,5]-hydrogen shift and an alternative [1,6]-hydrogen atom transfer from the 2-position of the sugar is known to be disfavored in the presence of acetyl protection [23]. The only available pathway for conformer **10-anti** is a radical disproportionation reaction leading to **7** and **8**, of which the latter re-enters the reaction cycle, while the former accumulates (Scheme 2).

On the other hand, conformer **10-syn** possesses a suitable conformation for a [1,5]-radical translocation leading to **11-syn** intermediate. After oxidation of **11-syn**, in the presence of I_2_, the **12-syn** oxonium ion may exist in equilibrium with conformer **12-anti**, through rotation of the C^1′^-N^1^ bond, and also with a possible Vorbrüggen-type intermediate, formed through the anchimeric assistance of the 2′-acetyl group. The Vorbrüggen intermediate is the main species that determines the stereochemistry of the final product in *N*-glycosylation reactions [28], and if the same was true in our system, exclusive formation of the *S*-anomer **9b** would be expected. Nevertheless, in our system, a 1:1.25 *S:R* mixture of anomers **9b**:**9a** was obtained. This result allows us to draw two major conclusions regarding the mechanism. First, rotation around the C^1′^-N^1^ bond in the oxonium ion **12-syn** to produce **12-anti** has to be faster than cyclization in order to allow the formation of the second, prior to cyclization, and there must be no major thermodynamic difference between the two conformers. Second, the formation of the Vorbrüggen intermediate must not be favored in this system, for steric reasons, and even if it is formed, through conformer **12-anti**, the process rate is comparable to that of the cyclization of **12-syn** conformer to the observed *S-*anomer **9a** (Scheme 2).

Kinetics determined that the *S* anomeric spirocycle **4b** exhibited 1.25 times higher inhibition than the *R* anomer **4a**. The difference could be associated with the locked *syn* conformation of the pyrimidine ring with regard to the β-d-glucose moiety and possible unfavorable interactions of **4a** within the catalytic site between the backbone CO of His377 with the uracil 2-C=O, as has been observed previously with protein crystallography (unpublished results). Although we attempted to obtain X-ray crystallographic data by soaking crystals of RMGP*b* with either **4a** or **4b**, the rather low affinity of both spirocycles did not provide sufficient data for establishing their binding in the catalytic site and studying their interactions. Both spironucleosides are stronger binders than the natural inhibitors of GP, β- and α-d-glucose [29]. For example, **4b** is about 7 and 1.5 times stronger than β- and α-d-glucose, respectively. The new compounds, nevertheless, exhibit a rather low inhibition profile compared with the known spirohydantoin derivative of glucopyranose [5] and other known strong catalytic site inhibitors of RMGP*b* [7,30]. We established previously that *anti* is the desirable conformation of the pyrimidine ring at the anomeric position of β-d-glucose leading to strong inhibition [7], and the current results confirm this finding. Anomeric spironucleosides are rigid structures and, given that they possess the correct conformation, are expected to bind strongly to the catalytic site of GP. Our current studies are therefore directed towards anomeric spironucleosides with locked *anti* conformations, and these results will be reported in due course.

## 4. Materials and Methods

All reagents and solvents were purchased from commercial sources (Sigma–Aldrich, Merck, NJ, USA; Alfa-Aesar, Fisher Scientific, MA, USA) and used without further purification, unless otherwise stated. All reactions were carried out under an argon atmosphere on a magnetic stirrer (IKA^®^-Werke GmbH & Co. KG, Staufen, Germany) and monitored by thin-layer chromatography. Compounds were purified by flash chromatography on silica gel 40–60 μm, 60 Å. NMR measurements were performed with a Varian Mercury 200 Nuclear Magnetic Resonance Spectrometer (Varian Inc., Agilent Technologies, Palo Alto, CA, USA) at 200 MHz for ^1^H and at 50 MHz for ^13^C, respectively. The deuterated solvents used for NMR spectroscopy were CDCl_3_ and D_2_O. Chemical shifts are given in ppm and were referenced on residual solvent peaks for CDCl_3_ (δ 7.26 ppm for ^1^H-NMR and 77.16 ppm for ^13^C-NMR), whereas for D_2_O an external reference of 3-(trimethylsilyl)-1-propanesulfonic acid sodium salt was used. Coupling constants were measured in Hz. Hydrogen atom assignments, when given, are based on COSY spectra. Melting points were obtained by using a Gallenkamp Sanyo apparatus (Fisher Scientific, MA, USA) and are uncorrected. Mass spectrometry experiments were carried out in a Thermo Finnigan Surveyor MSQ plus Mass Spectrometer (ThermoFisher Scientific, MA, USA), using the Electron Spray Ionization technique (ESI-MS). High-resolution mass spectrometry experiments were carried out in a Q-TOF Bruker MaXis Impact HR-Mass Spectrometer (Bruker, MA, USA). 1,2,3,4,6-Penta-*O*-acetyl-β*-*d*-*glucopyranose was synthesized using standard synthetic protocols [31]. AMP, Glc-1-P (dipotassium salt), and oyster glycogen were obtained from Sigma-Aldrich (Merck, NJ, USA) and used without further purification. Oyster glycogen was freed of AMP according to Helmreich and Cori [32]. ^1^H and ^13^C-NMR spectra of new compounds are available in the Supplementary Materials.

*2,4-Di-(trimethylsilyloxy)-(6-methylpyrimidine)* (**5**). A suspension of 6-methyluracil (1 g, 7.93 mmol) and well-grinded ammonium sulphate (80 mg, 0.60 mmol 0.076 eq) in HMDS (8.4 mL, 39.7 mmol, 5 eq) was heated to 120 °C under anhydrous conditions until full dissolution occurred. Upon completion, the excess of HMDS was removed through distillation, toluene was added twice (5 mL) followed by distillation to remove all traces of excess HMDS to yield 2.1 g (7.8 mmol, 97%) of the title compound which was characterized without further purification. ^1^H-NMR (200 MHz, CDCl_3_): δ = 0.34 (s, 18H), 1.95 (3H, s), 5.81 ppm (1H, s). ^13^C-NMR (50 MHz, CDCl_3_): δ = 0.00 (3C), 0.03 (3C), 23.4, 102.6, 162.6, 169.8, 170.0 ppm.

*N-Glycosylation of 6-methyluridine*. To a solution of **5** (1.5 g, 5.6 mmol, 1.5 eq) in dry 1,2-dichloroethane (7 mL) at r.t., a solution of TMSOTf (1.61 mL, 8.33 mmol, 2.25 eq) and 2,3,4,6-tetra-*O*-acetyl-β-d-glucopyranose[31] (1.44 g, 3.7 mmol) in dry DCE (3.5 mL) was added. The reaction mixture was heated at reflux until full consumption of the sugar (≈1 h). The mixture was then cooled, diluted with DCM, and washed successively twice with saturated aq. NaHCO_3_ solution, water, and brine. The organic layer was dried over anhydrous Na_2_SO_4_, filtered, the solvents evaporated, and the crude product was purified by column chromatography (30–70% Et_2_O in EtOAc) to give, in order of elution, **6c** as a white solid (86 mg, 0.11 mmol, 3%), **6a** as a white solid (1.13 g, 2.48 mmol, 67%) and **6b** as a white solid (0.51 g, 1.11 mmol, 30%).

*1-(Tetra-O-acetyl-β-d-glucopyranosyl)-6-methyluracil (***6a**): R_f_ = 0.40 (70:30 Et_2_O:EtOAc). ^1^H-NMR (200 MHz, CDCl_3_): δ = 2.00 (s, 3H), 2.02 (s, 3H), 2.06 (s, 3H), 2.08 (s, 3H), 2.55 (s, 3H), 3.90 (bd, *J* = 9.5 Hz, 1H), 4.16 (dd, 1H, *J* = 12.2, 1.7 Hz), 4.27 (dd, 1H, *J* = 12.6, 4.2 Hz), 5.14 (t, 1H, *J* = 9.5 Hz), 5.35 (t, 1H, *J* = 9.5 Hz), 5.46 (t, 1H, *J* = 9.4 Hz), 5.57 (bs, 2H), 6.27 (d, 1H, *J* = 9.1 Hz), 8.62 ppm (bs, 1H). ^13^C-NMR (50 MHz, CDCl_3_): δ = 20.60, 20.78, 20.80 (2C), 20.97, 61.8, 67.9, 69.5, 72.8, 75.1, 80.5, 104.1, 139.3, 150.5, 162.7, 169.7, 169.8, 170.0, 170.7 ppm. HRMS (ESI): calcd. for C_19_H_25_N_2_O_11_^+^ [M + H]^+^ 457.1458 found 457.1465.

*3-(Tetra-O-acetyl-β-d-glucopyranosyl)-6-methyluracil (***6b**): R_f_ = 0.35 (70:30 Et_2_O:EtOAc). ^1^H-NMR (200 MHz, CDCl_3_): δ = 1.95 (s, 3H), 2.01 (s, 3H), 2.04 (s, 3H), 2.06 (s, 3H), 2.17 (s, 3H), 3.84 (ddd, *J* = 10.1, 5.3, 2.5 Hz, 1H), 4.30–4.10 (m, 2H), 5.15 (t, *J* = 9.7 Hz, 1H), 5.30 (dd, *J* = 9.4, 8.6 Hz, 1H), 5.52 (s, 1H), 6.08 (d, *J* = 9.4 Hz, 1H), 6.18 (dd, *J* = 9.4, 8.4 Hz, 1H), 9.70 ppm (s, 1H). ^13^C-NMR (50 MHz, CDCl_3_): δ = 18.6, 20.4, 20.5 (2C), 20.6, 62.0, 67.9, 68.0, 73.7, 74.5, 78.3, 98.9, 151.5, 152.1, 162.5, 169.4, 169.6, 169.9, 170.5 ppm. ESI-MS: 457.2 [M + H]^+^.

*1,3-Bis-(tetra-O-acetyl-β-d-glucopyranosyl)-6-methyluracil* (**6c**): R_f_ = 0.50 (70:30 Et_2_O:EtOAc). ^1^H-NMR (200 MHz, CDCl_3_): δ = 2.01 (s, 6H), 2.02 (s, 6H), 2.04 (s, 6H), 2.06 (s, 6H), 2.57 (s, 1H), 3.96–3.74 (m, 2H), 4.37–4.10 (m, 4H), 5.52–5.02 (m, 5H), 5.55 (s, 1H), 6.02 (dd, *J* = 9.3, 9.3 Hz, 1H), 6.10 (d, *J* = 9.3 Hz, 1H), 6.34 ppm (d, *J* = 9.9 Hz, 1H). ^13^C-NMR (50 MHz CDCl_3_): δ = 20.0, 20.3, 20.6 (4C), 20.7 (3C), 61.4, 61.9, 67.5, 67.8, 68.1, 69.2, 73.3, 73.5, 74.7, 75.0, 79.2, 81.3, 103.4, 150.5, 152.9, 160.8, 169.40, 169.49, 169.62, 169.96, 170.20, 170.40, 170.60, 170.78 ppm. ESI-MS: 786.3 [M + H]^+^.

*1-(Tetra-O-acetyl-β-d-glucopyranosyl)-6-formyluracil* (**7**). To a solution of **6a** (2.1 g, 4.63 mmol) in dry dioxane (40 mL), selenium oxide was added (1.5 g, 13.9 mmol, 3 eq) and acetic acid (1.32 mL, 23.1 mmol). The reaction mixture was heated at reflux until full consumption of the starting material (≈5 h). The mixture was then cooled, diluted with ethyl acetate, and washed successively with saturated aq. NaHCO_3_ solution, water, and brine. The organic layer was dried over anhydrous Na_2_SO_4_, filtered, the solvents evaporated, and the crude product was purified by column chromatography (97:3, EtOAC:Et_2_O) to yield the title compound as a white solid (1.5 g, 3.1 mmol, 67%). R_f_ = 0.50 (100% EtOAc). ^1^H-NMR (200 MHz, CDCl_3_): δ = 2.01 (s, 3H), 2.04 (s, 3H), 2.06 (s, 3H), 2.10 (s, 3H), 3.92 (ddd, *J* = 9.9, 3.3, 3.0 Hz, 1H), 4.19 (m, 2H), 5.24 (t, *J* = 9.7 Hz, 1H), 5.41 (t, *J* = 9.4 Hz, 1H), 5.61 (t, *J* = 9.3 Hz, 1H), 6.11 (d, *J* = 9.4 Hz, 1H), 6.28 (d, *J* = 2.3 Hz, 1H), 8.53 (s, 1H), 9.90 ppm (s, 1H). ^13^C-NMR (50 MHz, CDCl_3_): δ = 20.2, 20.4 (2C), 20.5, 61.0, 67.2, 70.3, 72.2, 74.9, 81.3, 110.0, 147.0, 150.2, 161.6, 169.4, 169.7, 170.0, 170.4, 183.4 ppm. HRMS (ESI): calcd. for C_19_H_23_N_2_O_12_^+^ [M + H]^+^ 471.1251 found 471.1255.

*1-(Tetra-O-acetyl-β-d-glucopyranosyl)-6-hydroxymethyluracil* (**8**). To a solution of **7** (0.2 g, 0.425 mmol) in 2-propanol (2.5 mL) and chloroform (0.6 mL), dry silica gel was added (43 mg) and the suspension was cooled to −30 °C. Then, ΝaBH_4_ (0.161 g, 4.25 mmol, 10 eq) was added and the reaction mixture was stirred until full consumption of the starting material (≈1 h). Then, the mixture was diluted with DCM, filtered through Celite^®^, and the filtrate washed successively with saturated aq. NaHCO_3_ solution, water, and saturated sodium chloride solution. The organic layer was dried over anhydrous Na_2_SO_4_, filtered, the solvents were evaporated, and the crude product was purified by column chromatography (EtOAc) to give the title compound as a white solid (146 mg, 0,38 mmol, 90%). R_f_ = 0.40 (70:30, Et_2_O:EtOAc). ^1^H-NMR (200 ΜHz, DMSO-d6): (mixture of tautomers) δ = 1.92 (s, 3H), 1.96 (s, 3H), 2.01 (s, 6H), 4.30 (m, 2H), 4.92 (t, *J* = 9.6 Hz, 1H), 5.26–5.62 (m, 2H), 5.69 (s, 1H), 5.84 (s, 2H), 6.08 (t, *J* = 9.0 Hz, 1H), 6.42 (d, *J* = 9.4 Hz, 1H), 11.32 ppm (s, 1H), 11.48 ppm (s, 1H). ^13^C-NMR (50 MHz, CDCl_3_): δ = 20.4, 20.5 (2C), 20.7, 60.1, 61.2, 67.5, 69.8, 72.6, 75.2, 81.5, 102.9, 151.3, 157.2, 162.8, 169.5, 169.8, 170.4, 170.7 ppm. HRMS (ESI): calcd. for C_19_H_25_N_2_O_12_^+^ [M + H]^+^ 473.1407 found 473.1410.

Spirocyclization of **8**. A solution of **8** (150 mg, 0.32 mmol) in dichloromethane (16 mL) was degassed by argon gas bubbling for 10 min. Then, diacetoxyiodobenzene (155 mg, 0.48 mmol, 1.5 eq) and iodine (91 mg, 0.32 mmol, 1 eq) were added. Photolysis was carried out at r.t., with two 75 W Philips Standard 230 V visible light lamps, for 2.5 h. Afterwards, the reaction was quenched by 10% aq. Na_2_S_2_O_3_ solution and then extracted with dichloromethane. The organic layer was collected, dried over anhydrous Na_2_SO_4_, and then filtered, the solvent was evaporated, and the crude product was purified by column chromatography (EtOAc: Et_2_O gradient) to give, in order of elution, compound **7** (75 mg, 0.16 mmol, 50%), **9a** as a white solid, (12 mg, 0.026 mmol, 8%), and **9b** as a white solid (15 mg, 0.032 mmol, 10%).

(3*R*,3′*R*,4′*S*,5′*R*,6′*R*)-6′-(Acetoxymethyl)-5,7-dioxo-1,3′,4′,5,5′,6,6′,7-octahydrospiro[oxazolo[3,4-*c*]pyrimidine-3,2′-pyran]-3′,4′,5′-triyl triacetate (**9a**): R_f_ = 0.60 (70:30 Et_2_O:EtOAc). ^1^H-NMR (200 MHz, CDCl_3_): δ = 2.00 (s, 3H), 2.01 (s, 3H), 2.05 (s, 3H), 2.09 (s, 3H), 4.17–4.10 (m, 1H), 4.20 (s, 2H), 5.04 (m, 2H), 5.34 (t, *J* = 9.5 Hz, 1H), 5.48 (t, *J* = 10.0 Hz, 1H), 5.59 (s, 1H), 6.16 ppm (d, *J* = 9.8 Hz, 1H). ^13^C-NMR (50 MHz, CDCl_3_): δ = 20.52, 20.60, 20.62, 20.72, 61.2, 67.4, 68.2, 68.6, 71.3, 71.4, 93.8, 112.1, 145.7, 151.3, 163.1, 168.7, 169.2, 170.1, 170.7 ppm. HRMS (ESI): calcd. for C_19_H_23_N_2_O_12_^+^ [M + H]^+^ 471.1246 found 471.1239.

(3*S*,3′*R*,4′*S*,5′*R*,6′*R*)-6′-(Acetoxymethyl)-5,7-dioxo-1,3′,4′,5,5′,6,6′,7-octahydrospiro[oxazolo[3,4-*c*]pyrimidine-3,2′-pyran]-3′,4′,5′-triyl triacetate (**9b**): R_f_ = 0.40 (70:30 Et_2_O:EtOAc). ^1^H-NMR (200 MHz, CDCl_3_): δ = 1.99 (s, 3H), 2.03 (s, 3H), 2.05 (s, 3H), 2.09 (s, 3H), 4.10 (dd, *J* = 12.7, 2.5 Hz, 1H), 4.26 (dd, *J* = 12.7, 3.6 Hz, 1H), 4.65 (ddd, *J* = 9.6, 3.6, 2.5 Hz, 1H), 4.87 (dd, *J* = 14.5, 1.0 Hz, 1H), 5.06 (dd, *J* = 14.5, 1.6 Hz, 1H), 5.33 (dd, *J* = 10.1, 8.9 Hz, 1H), 5.63 (d, *J* = 9.3 Hz, 1H), 5.65 (s, 1H), 6.31 (t, *J* = 9.2 Hz, 1H), 8.61 ppm (s, 1H). ^13^C-NMR (50 MHz, CDCl_3_): δ = 20.52, 20.60, 20.62, 20.70, 61.4, 67.2, 67.3, 71.0, 72.4, 72.6, 94.2, 114.5, 148.3, 152.7, 163.3, 169.1, 169.6, 170.0, 170.6 ppm. HRMS (ESI): calcd. for C_19_H_23_N_2_O_12_^+^ [M + H]^+^ 471.1246 found 471.1241.

(3*R*,3′*R*,4′*S*,5′*S*,6′*R*)-3′,4′,5′-Trihydroxy-6′-(hydroxymethyl)-3′,4′,5′,6′-tetrahydrospiro[oxazolo[3,4-*c*]pyrimidine-3,2′-pyran]-5,7(1H,6H)-dione (**4a**). A solution of **9a** (20 mg, 0.043 mmol) in methanolic ammonia (7 N, 0.35 mL) was stirred for 12 h at r.t., until full conversion to a single compound. Then, the solvent was evaporated and the compound was dried under high vacuum to yield the title compound as a white solid (13 mg, 0.043 mmol, 100%). ^1^H-NMR (200 MHz, D_2_O): δ = 3.64 (t, *J* = 9.1 Hz, 1H), 3.97–3.75 (m, 4H), 4.56 (d, *J* = 9.7 Hz, 1H), 5.20 (s, 2H), 5.88 ppm (s, 1H). ^13^C-NMR (50 MHz, D_2_O): δ = 63.0, 70.8, 71.6, 72.5, 76.5, 78.3, 96.7, 116.8, 142.5, 157.1, 169.6 ppm. HRMS (ESI): calcd. for C_11_H_15_N_2_O_8_^+^ [M + H]^+^ 303.0823 found 303.0830.

(3*S*,3′*R*,4′*S*,5′*S*,6′*R*)-3′,4′,5′-Trihydroxy-6′-(hydroxymethyl)-3′,4′,5′,6′-tetrahydrospiro[oxazolo[3,4-*c*]pyrimidine-3,2′-pyran]-5,7(1H,6H)-dione (**4b**). A solution of **9b** (29 mg, 0.062 mmol) in methanolic ammonia (7 N, 0.51 mL) was stirred for 12 h at r.t., until full conversion to a single compound. Then, the solvent was evaporated and the compound was dried under high vacuum to yield the title compound as a white solid (19 mg, 0.062 mmol, 100%). ^1^H-NMR (200 MHz, D_2_O): δ = 3.58 (t, *J* = 9.6 Hz, 1H), 3.70 (dd, *J* = 12.5, 5.5 Hz, 2H), 3.85 (dd, *J* = 12.4, 2.1 Hz, 1H), 3.95 (d, *J* = 9.5 Hz, 2H), 4.26 (ddd, *J* = 10.1, 5.3, 2.2 Hz, 1H), 4.62 (t, *J* = 9.2 Hz, 2H), 5.08 (d, *J* = 14.9 Hz, 1H), 5.18 (d, *J* = 15.0 Hz, 1H), 5.81 ppm (s, 1H). ^13^C-NMR (50 MHz, D_2_O): δ = 169.5, 158.2, 149.4, 119.6, 96.4, 79.5, 77.3, 76.2, 71.6, 70.2, 63.4 ppm. HRMS (ESI): calcd. for C_11_H_15_N_2_O_8_^+^ [M + H]^+^ 303.0823 found 303.0828.

## 5. Conclusions

In conclusion, we successfully applied the Suárez radical methodology to synthesize the first example of a 1,6-dioxa-4-azaspiro[4.5]decane system starting from 6-hydroxymethyl-β-d- glucopyranosyluracil. We showed that, in the rigid pyranosyl conformation, the required [1,5]-radical translocation is a minor process. The stereochemistry of the spirocycles obtained was unequivocally determined by the chemical shifts of key sugar protons in the ^1^H-NMR spectra. Finally, the two spirocycles were found to be modest inhibitors of RMGP*b*, corroborating the finding that *anti* should be the desired conformation of the pyrimidine ring of future anomeric spironucleosides, which may lead to strong inhibition of GP.

## Supplementary Materials

The following are available online. ^1^H and ^13^C-NMR spectra of new compounds (Figures S1–10). Tables of kinetic measurements (Tables S1 and S2).
