# Supplementary material for: Anomeric Spironucleosides of β-d-Glucopyranosyl Uracil as Potential Inhibitors of Glycogen Phosphorylase
Source: Molecules. 2019 Jun 25;24(12):2327. doi: 10.3390/molecules24122327 (PMC6630470; doi:10.3390/molecules24122327)
Supplement: Supplementary file 1 [file molecules-24-02327-s001.pdf]

Anomeric spironucleosides of β-D-glucopyranosyl uracil as potential inhibitors of glycogen phosphorylase

Aggeliki Stathi[1], Michael Mamais[1,2], Evangelia D. Chrysina[2] and Thanasis Gimisis[1,*]

[1] *Organic Chemistry Laboratory, Department of Chemistry, National and Kapodistrian University of Athens, 10571, Athens, Greece.e-mail: gimisis@chem.uoa.gr.*

[2] *Institute of Biology, Medicinal Chemistry & Biotechnology, National Hellenic Research Foundation, Athens (Greece). e-mail: echrysina@eie.gr.*

Supplementary Materials

Contents

Figures S1-S21. NMR spectra of compounds **5, 6a-c, 7, 8, 9a,b** and **4a,b**. **2-12**

Tables S1-2. Enzyme kinetics for compounds **4a,b** and RMGP*b*. **13**

Figure S1. ^{1}H NMR spectrum of compound **5** (200 MHz, $CDCl_3$).

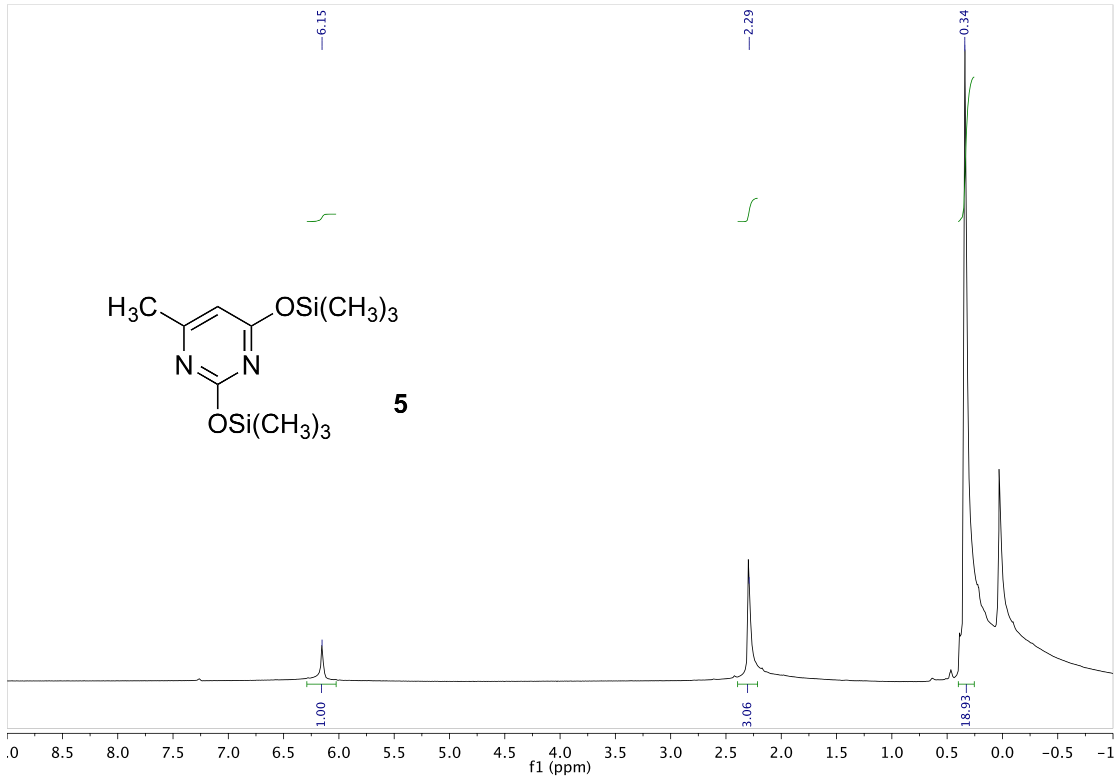

Figure S2. ^{13}C NMR spectrum of compound **5** (50 MHz, $CDCl_3$).

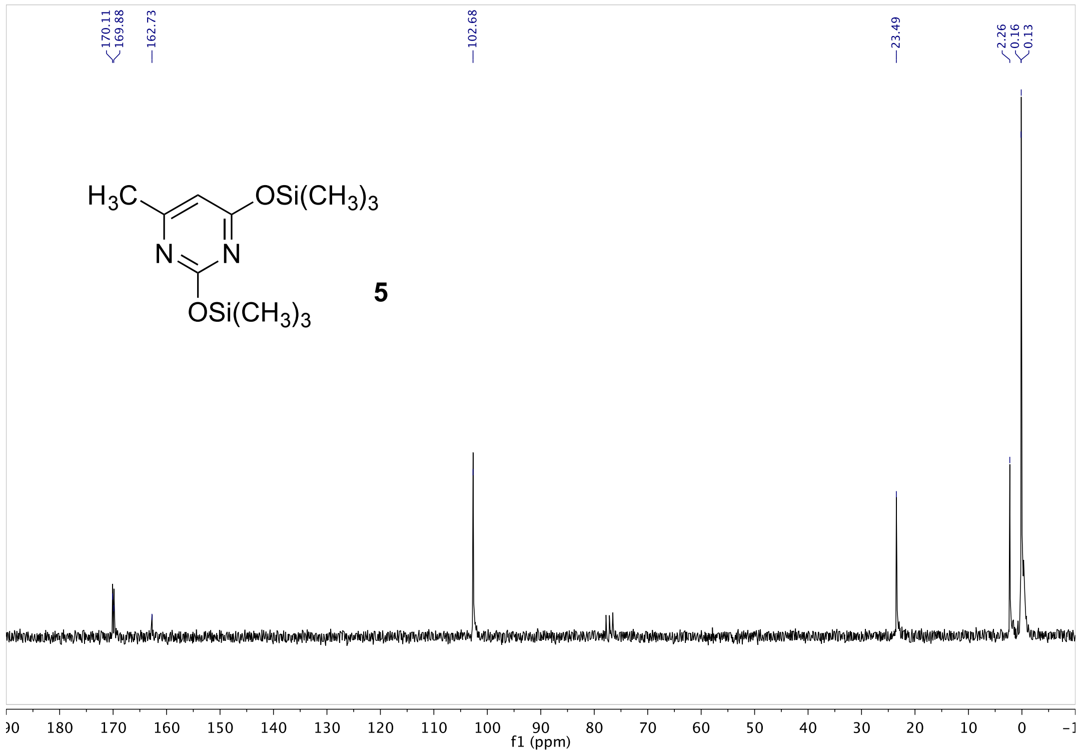

Figure S3. ^{1}H NMR spectrum of compound **6a** (200 MHz, $CDCl_3$).

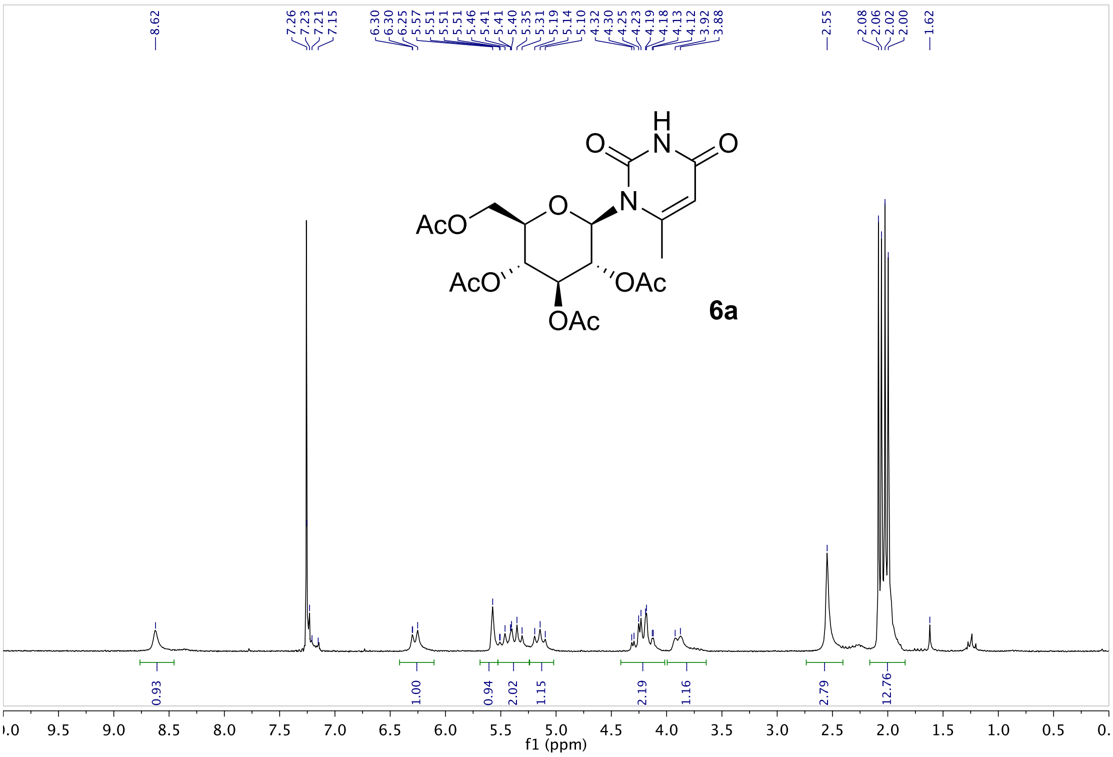

Figure S4. ^{13}C NMR spectrum of compound **6a** (50 MHz, $CDCl_3$).

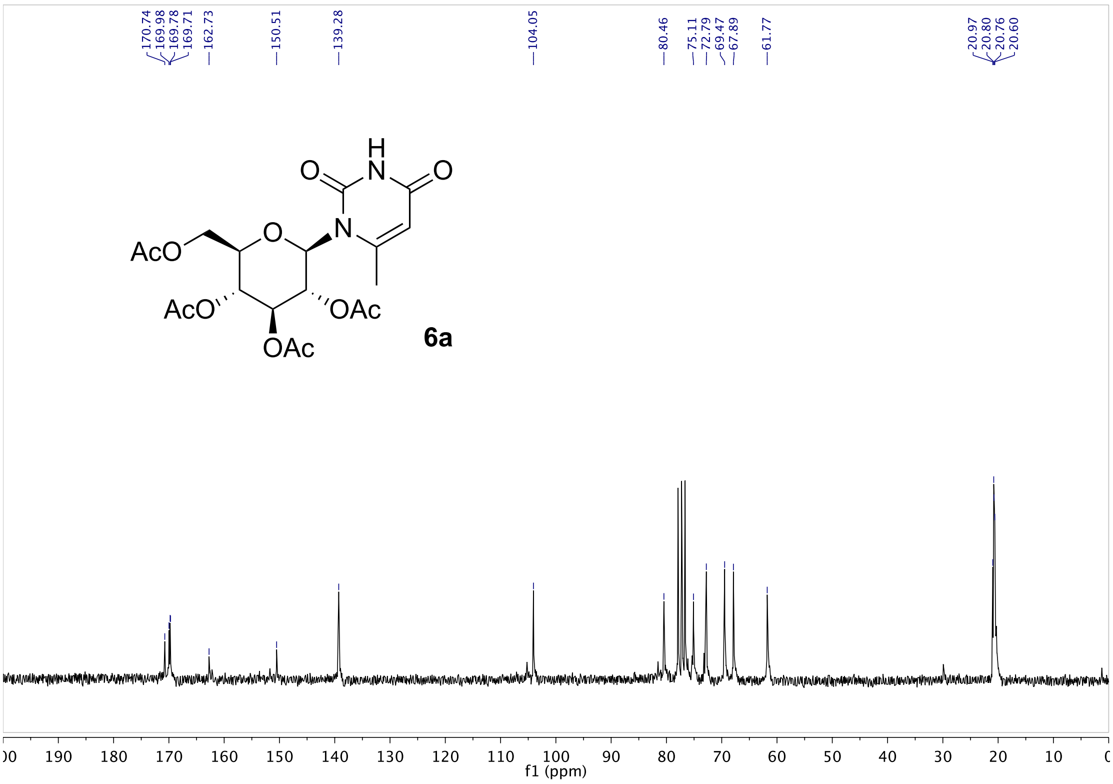

Figure S5. ^{1}H NMR spectrum of compound **6b** (200 MHz, $CDCl_3$).

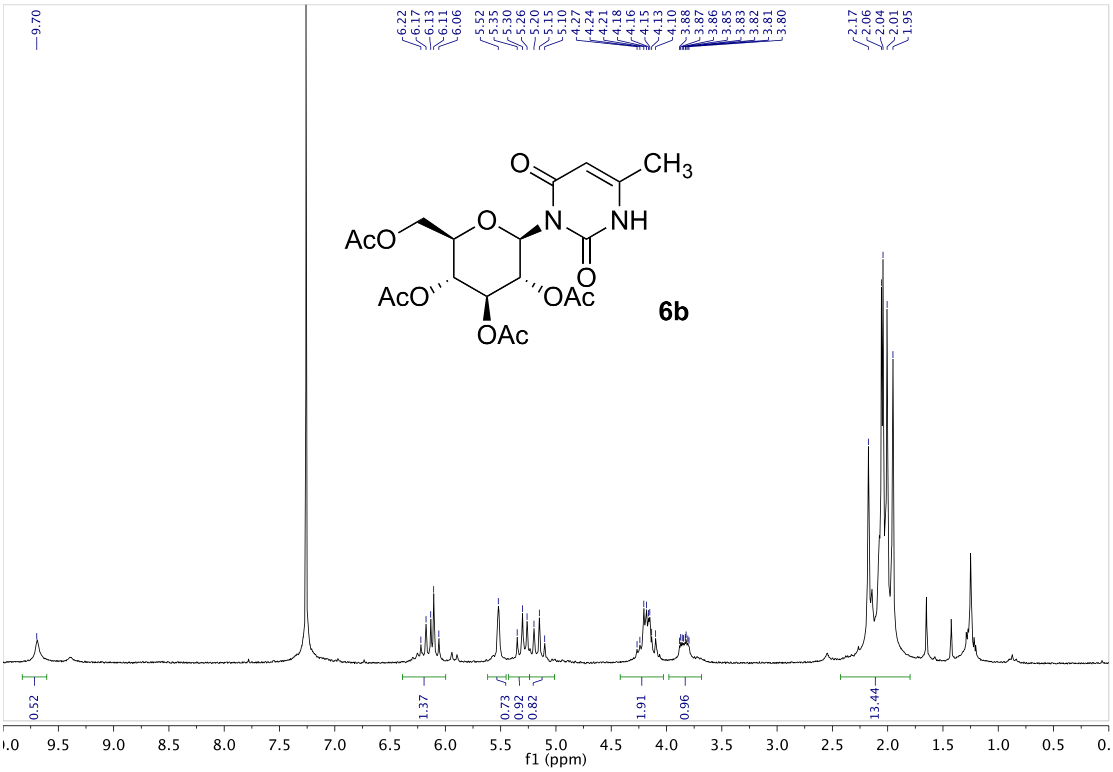

Figure S6. ^{13}C NMR spectrum of compound **6b** (50 MHz, $CDCl_3$).

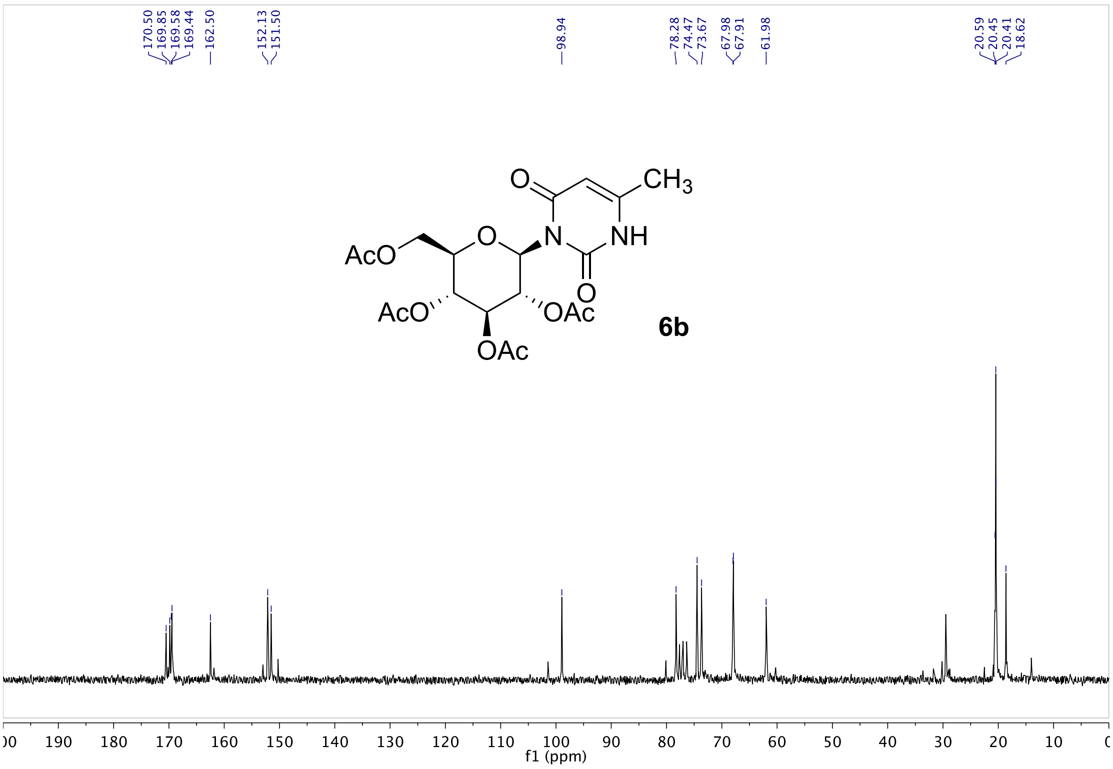

Figure S7. ^{1}H NMR spectrum of compound **6c** (200 MHz, $CDCl_3$).

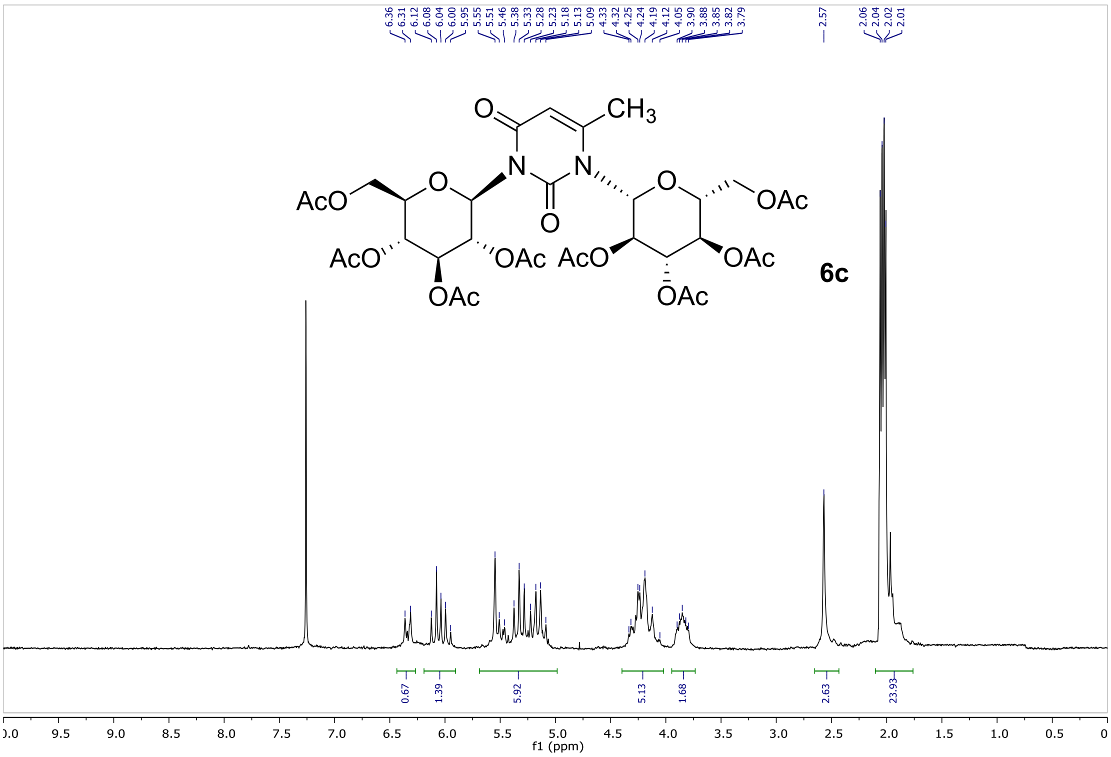

Figure S8. ^{13}C NMR spectrum of compound **6b** (50 MHz, $CDCl_3$).

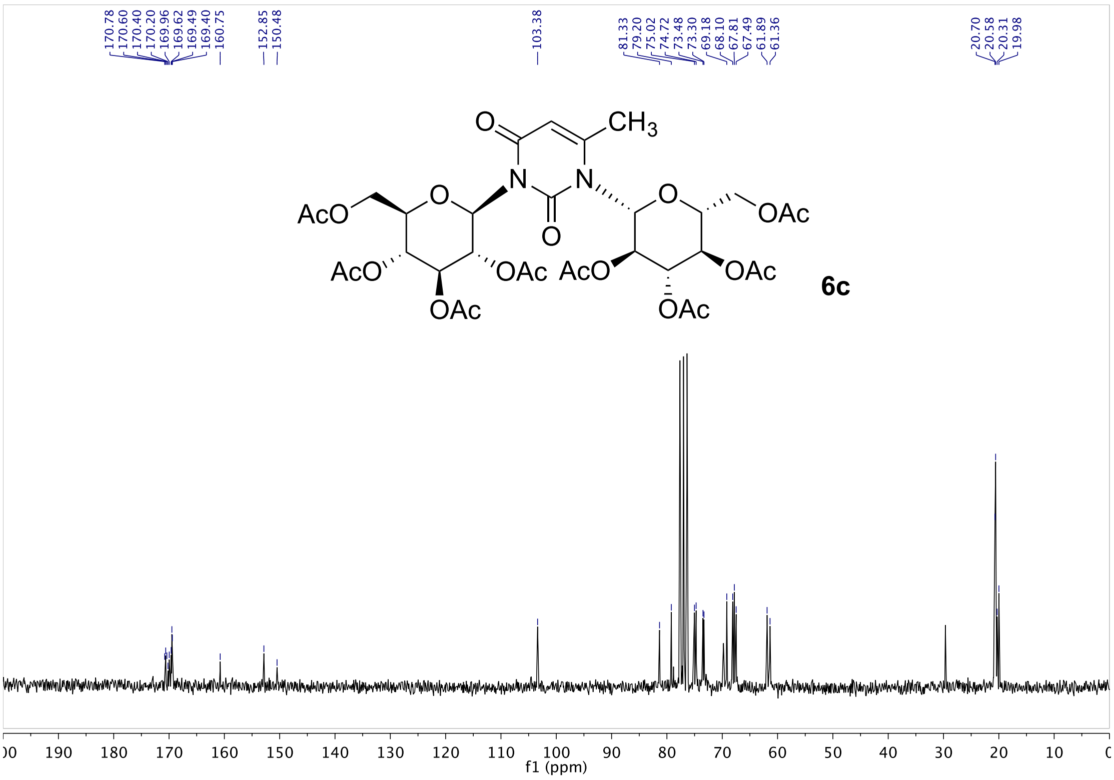

Figure S9. ^{1}H NMR spectrum of compound **7** (200 MHz, $CDCl_3$).

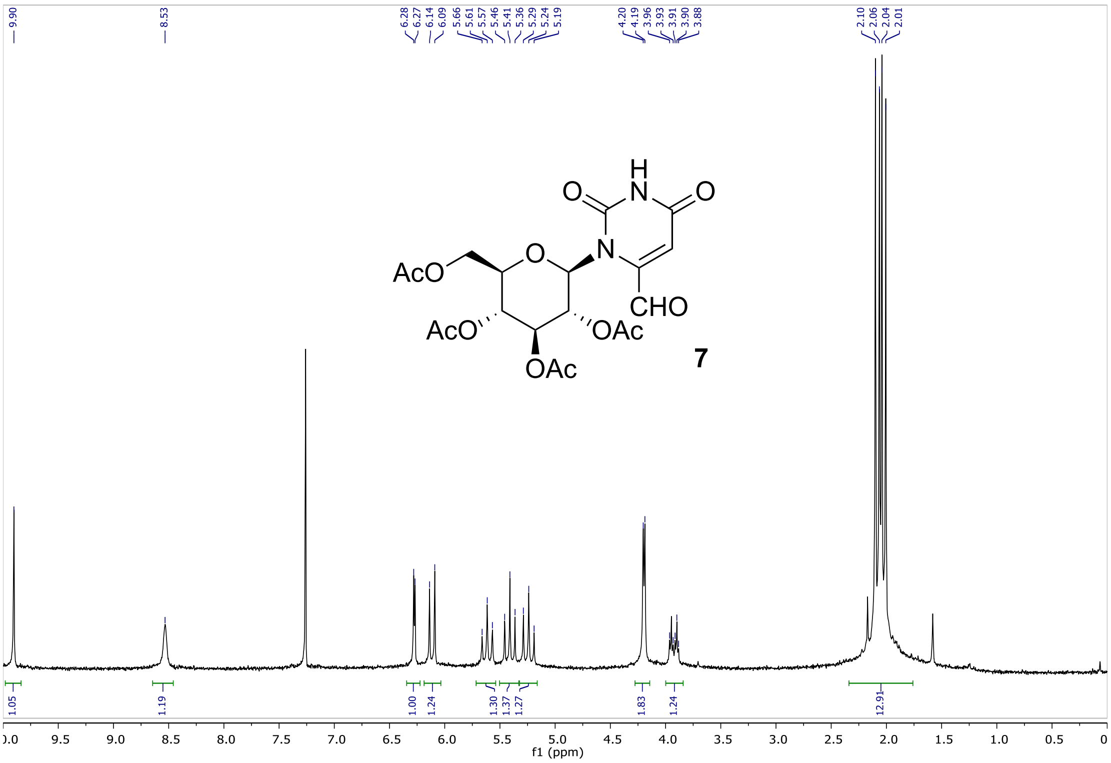

Figure S10. ^{13}C NMR spectrum of compound **7** (50 MHz, $CDCl_3$).

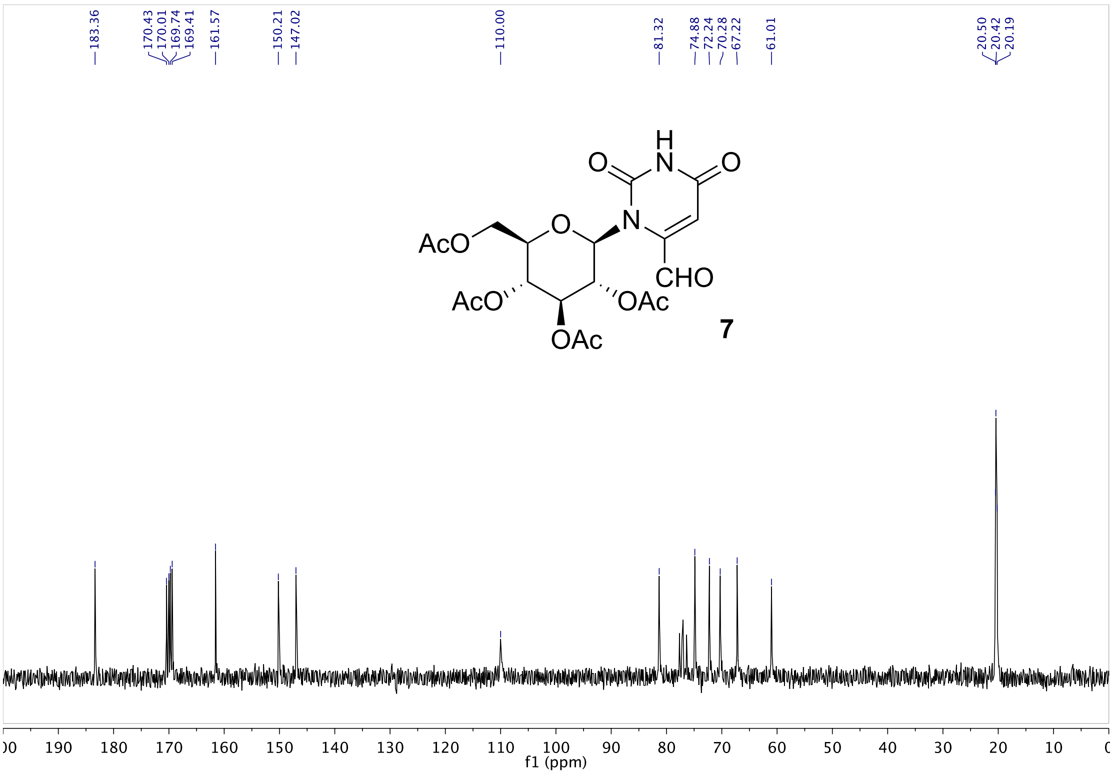

Figure S11. ^{1}H NMR spectrum of compound **8** (200 MHz, DMSO-d6).

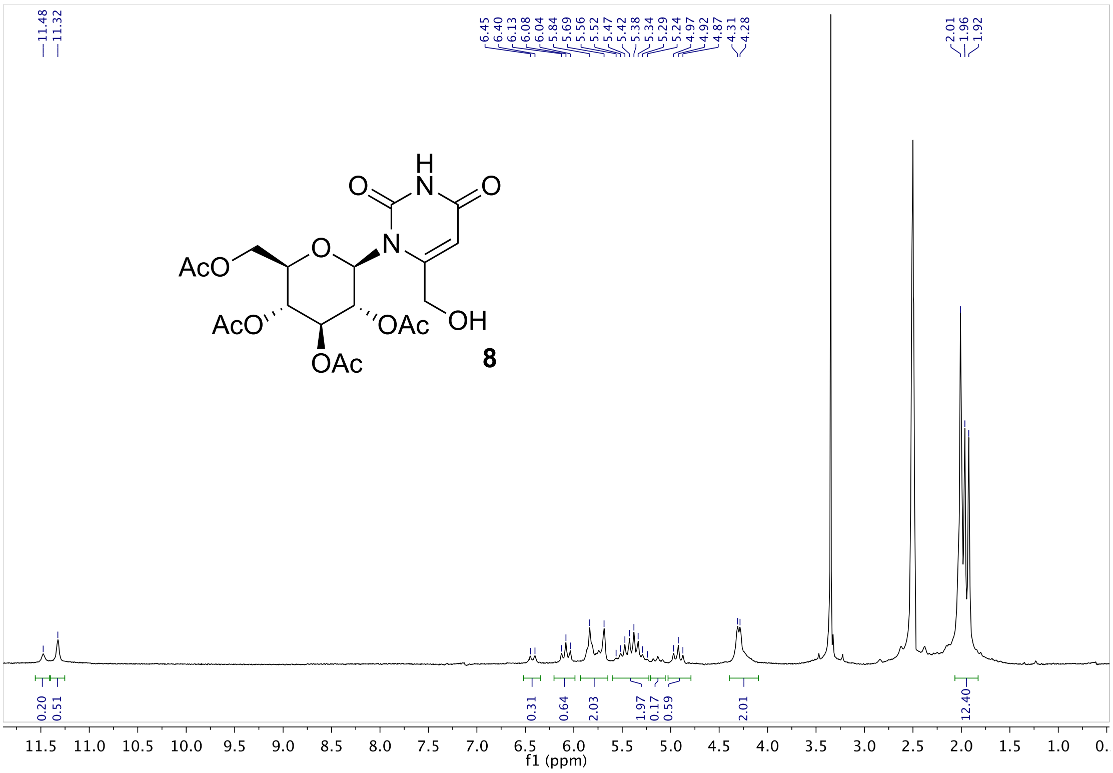

Figure S12. ^{1}H NMR spectrum of compound **8** (200 MHz, DMSO-d6) at 75 °C.

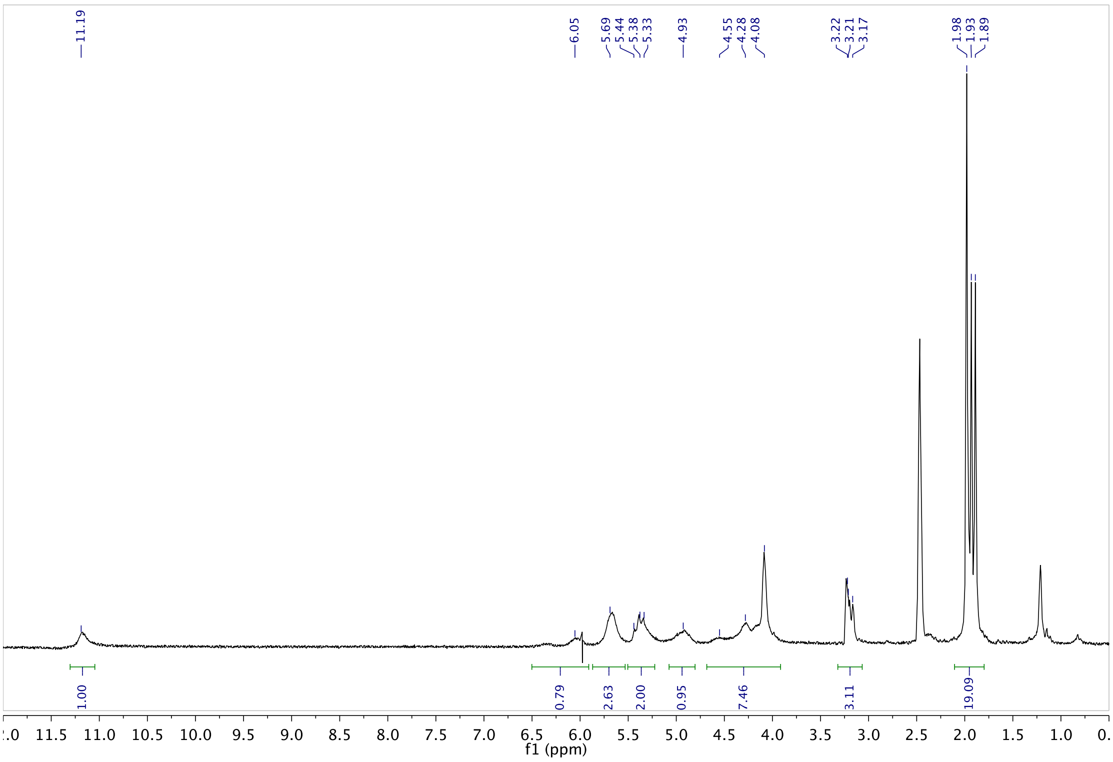

Figure S13. ^{13}C NMR spectrum of compound **8** (50 MHz, $CDCl_3$).

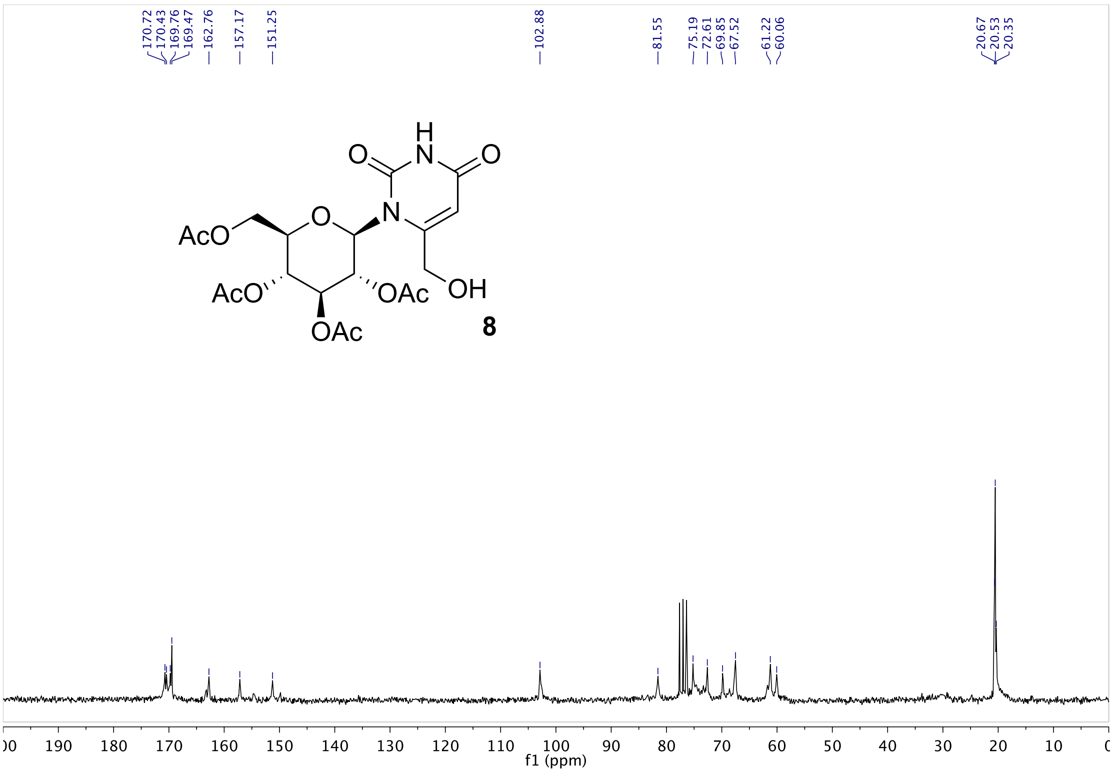

Figure S14. ^{1}H NMR spectrum of compound **9a** (200 MHz, $CDCl_3$).

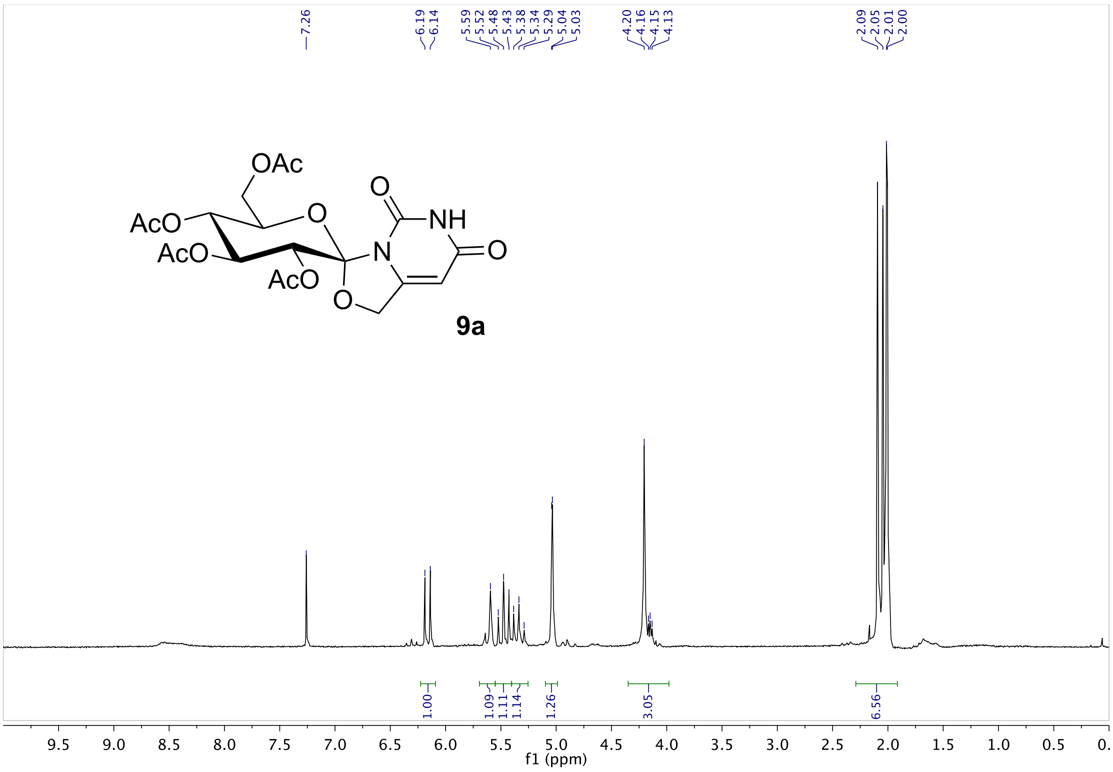

Figure S15. ^{13}C NMR spectrum of compound **9a** (50 MHz, $CDCl_3$).

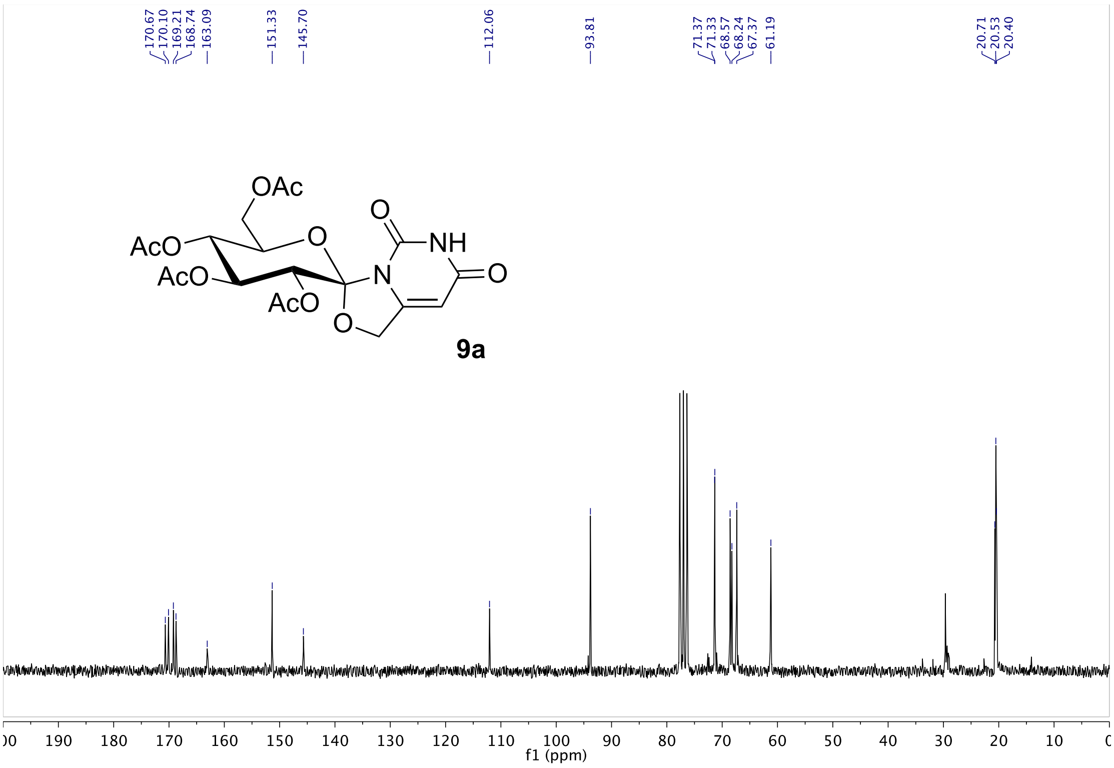

Figure S16. ^{1}H NMR spectrum of compound **9b** (200 MHz, $CDCl_3$).

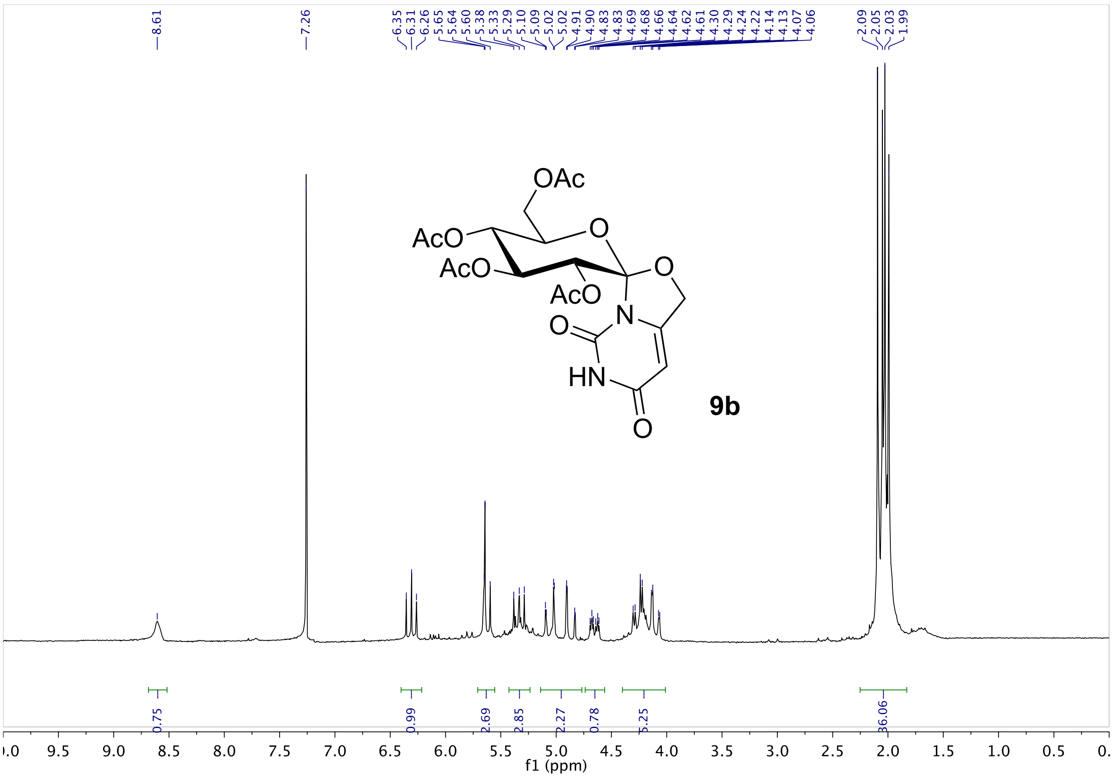

Figure S17. ^{13}C NMR spectrum of compound **9b** (50 MHz, $CDCl_3$).

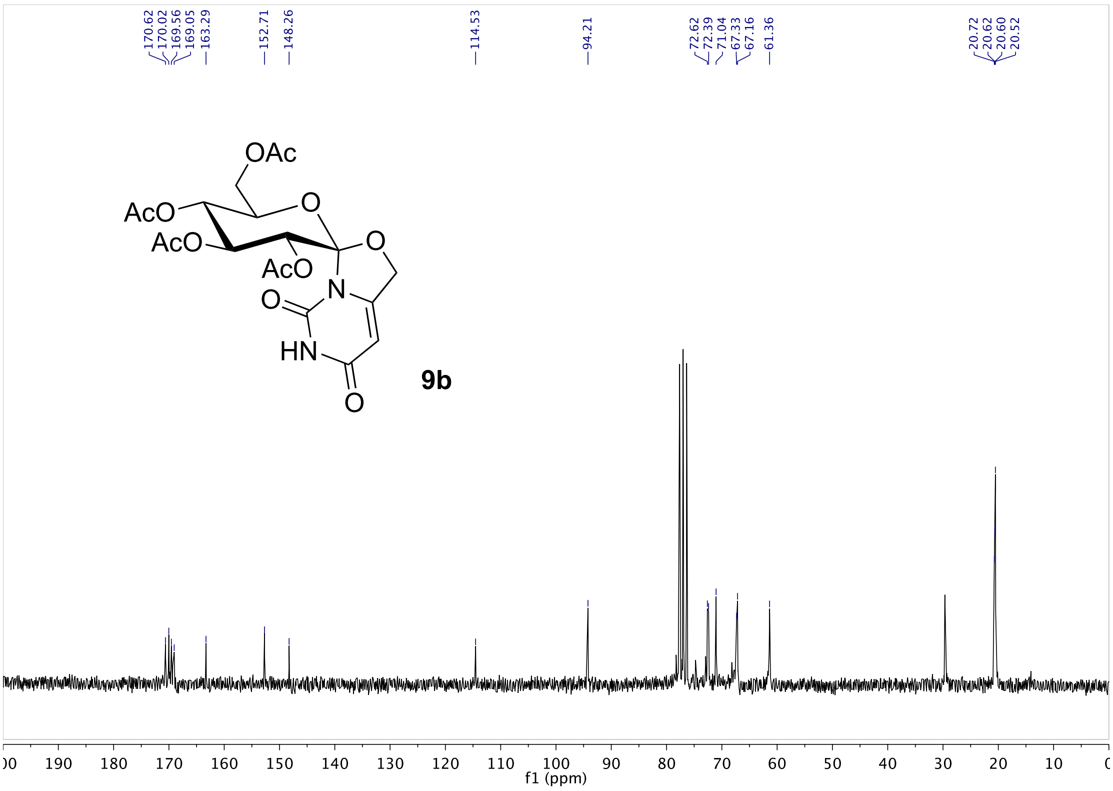

Figure S18. ^{1}H NMR spectrum of compound **4a** (200 MHz, $CDCl_3$).

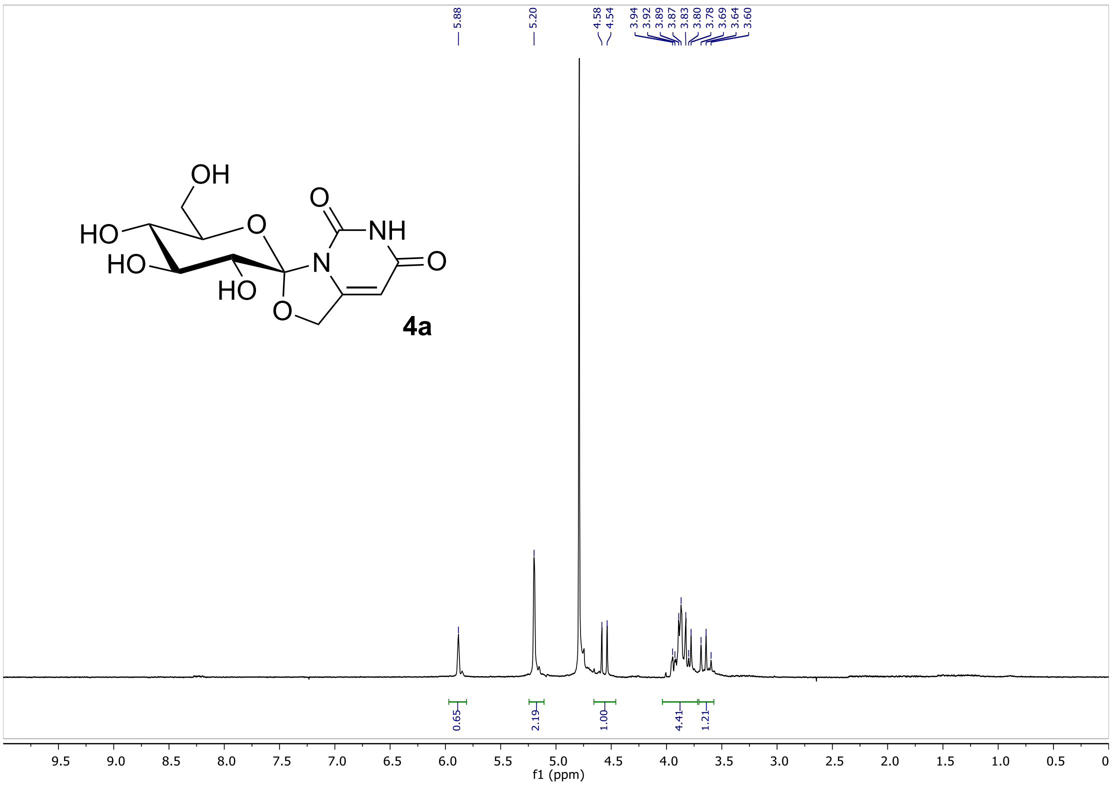

Figure S19. ^{13}C NMR spectrum of compound **4a** (50 MHz, $CDCl_3$).

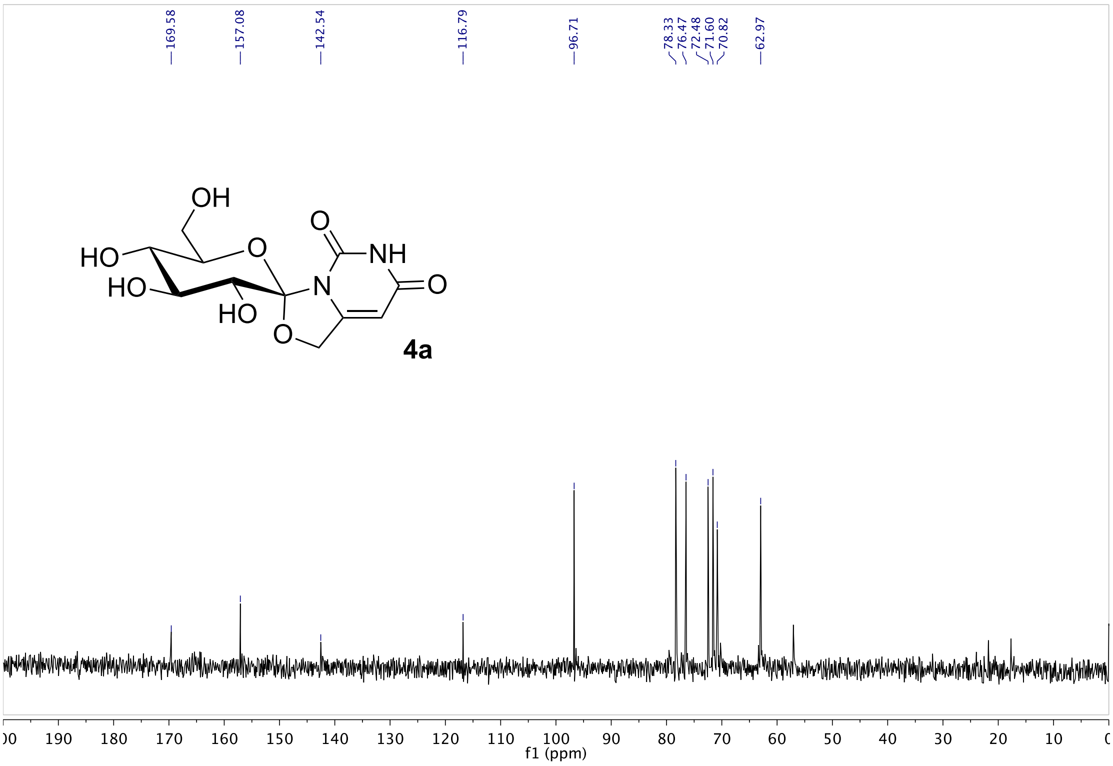

Figure S20. ^{1}H NMR spectrum of compound **4b** (200 MHz, D_2O).

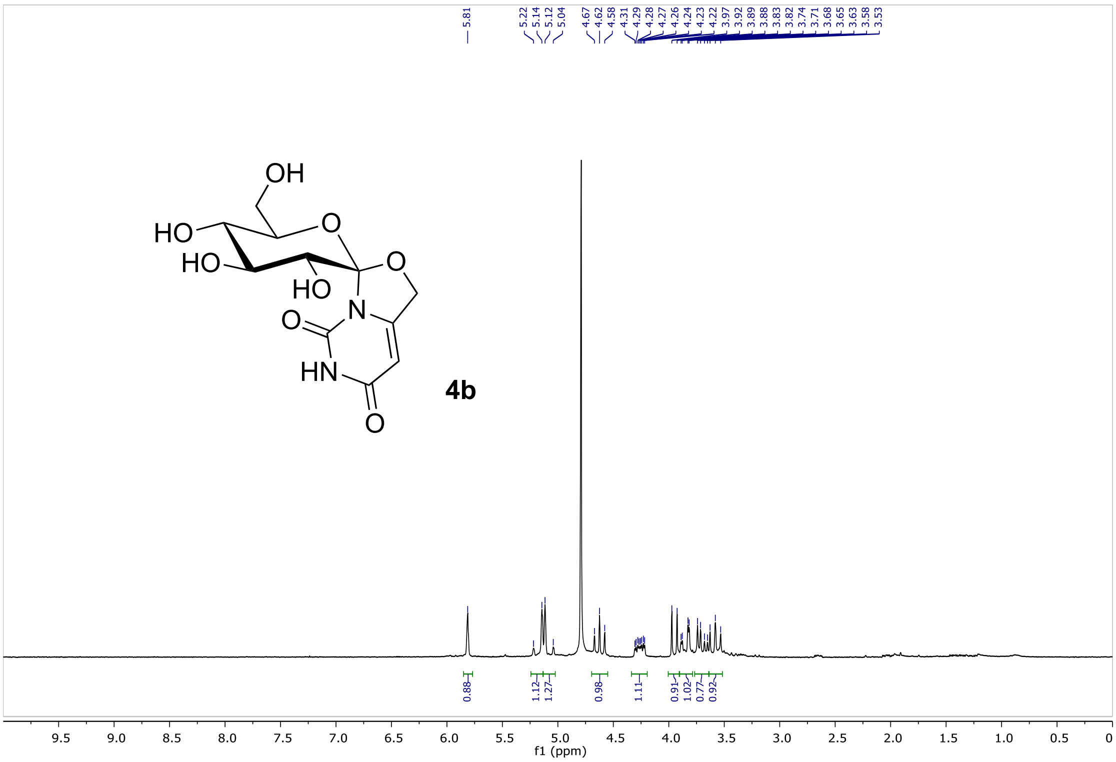

Figure S21. ^{13}C NMR spectrum of compound **4b** (50 MHz, D_2O).

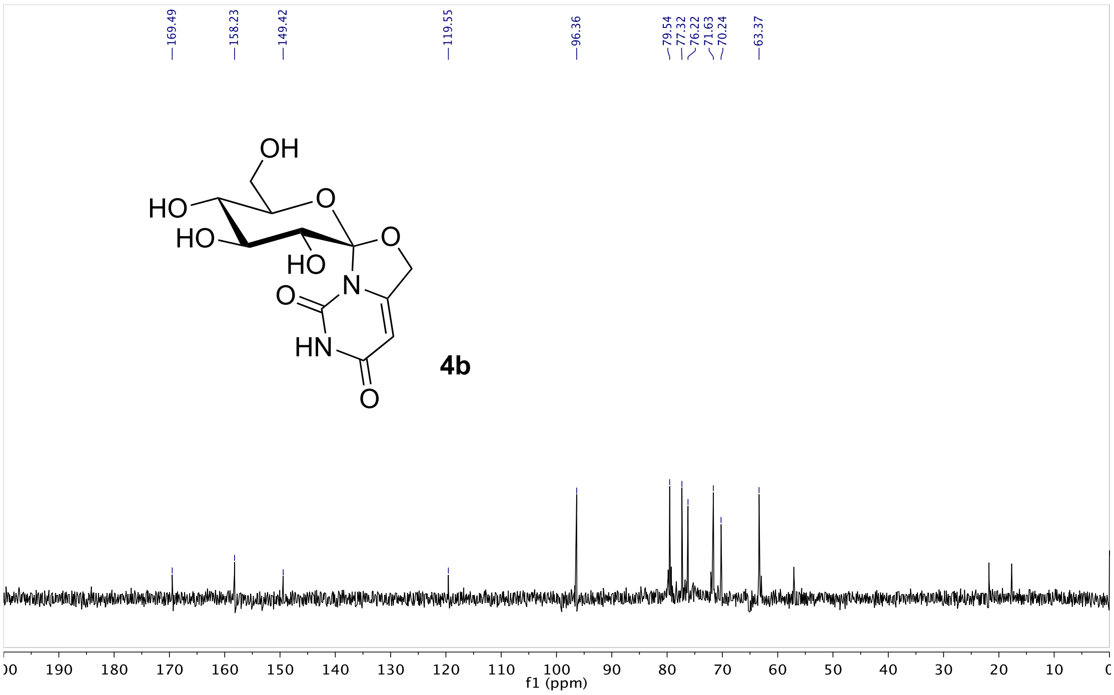

2. Enzyme Kinetics

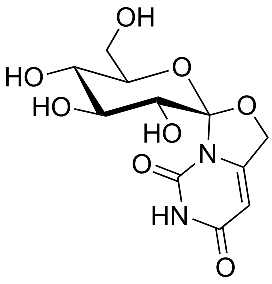

4b

RMGP*b* = 5 γ/ml
G1p = 2 mM
AMP = 1 mM
Gly = 0.2%
Buffer =
β-glycerophosphate:mercaptoethanol:EDTA
0.5 : 0.5 : 0.01
Km = 3 mM

Table S1. Inhibition vs **4b** concentration.

[4b] (μM)	% Inhibition
200	6.9
400	19.7
600	25.0
1000	35.0

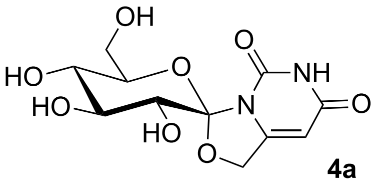

4a

RMGP*b* = 5 γ/ml
G1p = 2 mM
AMP = 1 mM
Gly = 0.2%
Buffer =
β-glycerophosphate:mercaptoethanol:EDTA
0.5 : 0.5 : 0.01
Km = 3 mM

Table S2. Inhibition vs **4a** concentration.

[4a] (μM)	% Inhibition
200	1.7
400	9.4
600	18.3
1000	25.8
